# Cerebellum in Levodopa-Induced Dyskinesias: The Unusual Suspect in the Motor Network

**DOI:** 10.3389/fneur.2014.00157

**Published:** 2014-08-18

**Authors:** Asha Kishore, Traian Popa

**Affiliations:** ^1^Department of Neurology, Comprehensive Care Centre for Movement Disorders, Sree Chitra Tirunal Institute for Medical Sciences and Technology, Kerala, India; ^2^Centre de Neuroimagerie de Recherche (CENIR), Institut du Cerveau et de la Moelleepiniere (ICM), Paris, France

**Keywords:** levodopa-induced dyskinesias, dopamine, Parkinson’s disease, plasticity, motor cortex, cerebellum, basal ganglia

## Abstract

The exact mechanisms that generate levodopa-induced dyskinesias (LID) during chronic levodopa therapy for Parkinson’s disease (PD) are not yet fully established. The most widely accepted theories incriminate the non-physiological synthesis, release and reuptake of dopamine generated by exogenously administered levodopa in the striatum, and the aberrant plasticity in the cortico-striatal loops. However, normal motor performance requires the correct recruitment of motor maps. This depends on a high level of synergy within the primary motor cortex (M1) as well as between M1 and other cortical and subcortical areas, for which dopamine is necessary. The plastic mechanisms within M1, which are crucial for the maintenance of this synergy, are disrupted both during “OFF” and dyskinetic states in PD. When tested without levodopa, dyskinetic patients show loss of treatment benefits on long-term potentiation and long-term depression-like plasticity of the intracortical circuits. When tested with the regular pulsatile levodopa doses, they show further impairment of the M1 plasticity, such as inability to depotentiate an already facilitated synapse and paradoxical facilitation in response to afferent input aimed at synaptic inhibition. Dyskinetic patients have also severe impairment of the associative, sensorimotor plasticity of M1 attributed to deficient cerebellar modulation of sensory afferents to M1. Here, we review the anatomical and functional studies, including the recently described bidirectional connections between the cerebellum and the basal ganglia that support a key role of the cerebellum in the generation of LID. This model stipulates that aberrant neuronal synchrony in PD with LID may propagate from the subthalamic nucleus to the cerebellum and “lock” the cerebellar cortex in a hyperactive state. This could affect critical cerebellar functions such as the dynamic and discrete modulation of M1 plasticity and the matching of motor commands with sensory information from the environment during motor performance. We propose that in dyskinesias, M1 neurons have lost the ability to depotentiate an activated synapse when exposed to acute pulsatile, non-physiological, dopaminergic surges and become abnormally receptive to unfiltered, aberrant, and non-salient afferent inputs from the environment. The motor program selection in response to such non-salient and behaviorally irrelevant afferent inputs would be abnormal and involuntary. The motor responses are worsened by the lack of normal subcortico–cortical inputs from cerebellum and basal ganglia, because of the aberrant plasticity at their own synapses. Artificial cerebellar stimulation might help re-establish the cerebellar and basal ganglia control over the non-salient inputs to the motor areas during synaptic dopaminergic surges.

## Introduction

In spite of being the most efficacious drug for the relief of motor symptoms of Parkinson’s disease (PD), l-3,4-dihydroxyphenylalanine (also known as levodopa) almost invariably generates disabling involuntary movements. Levodopa-induced dyskinesias (LID) seldom occur with the first dose of levodopa, but chronic exposure to the drug results in LID in 20–30% of PD patients in ∼2 years and in 80% within 5 years (1). The risk to develop LID is enhanced by younger age, longer durations of disease and levodopa treatment, greater disease severity (1), higher levodopa dose (2), genetic etiology of the disease (3–5) and genetic variability in the dopamine metabolizing enzymes (6), dopamine receptor and transporter isoforms (7–9), and brain derived neurotrophic factor (10).

Based on the timing of their appearance in a levodopa cycle, LID are termed as “peak-dose dyskinesia” or “biphasic dyskinesia.” Peak-dose dyskinesias are associated with high plasma concentrations of levodopa (11) and the maximum reduction in Parkinsonian signs. Biphasic dyskinesias appear just before the beginning and the end of the relief period of the Parkinsonian signs, disappear in the phase of maximum clinical response, and occur below a critical, low level of plasma levodopa (12). Peak-dose dyskinesias are often choreic and seldom pure dystonic movements. Biphasic dyskinesias are stereotyped, repetitive, dystonic movements that are usually confined to the legs, and may co-occur with Parkinsonian signs elsewhere in the body. Dystonic movements can also occur in patients not exposed to levodopa and their presence correlates with akinesia of PD (13).

The neural mechanisms of LID, those that determine the clinical type of LID (i.e., choreic or dystonic) and those allowing the co-occurrence of Parkinsonism and biphasic dyskinesias, are still not fully understood (13–16).

The most accepted models of LID implicate pre- and post-synaptic changes at the cortico-striatal synapses and alterations in the activity of dopaminergic and non-dopaminergic (e.g., glutamatergic) neurotransmitter systems. The degeneration of dopaminergic neurons in the substantia nigra pars compacta (SNc) is associated with a series of changes in the nigro-striatal synapses such as a loss of tonic release of dopamine, diminished dopamine storage, and reuptake capacity [for review see Ref. (17)]. Positron emission imaging studies have demonstrated abnormally high levels of striatal dopamine 1-hour post-medication (18) and reduced dopamine transporter (DAT) levels (19) in dyskinetic patients. Brain concentrations of levodopa after peripheral administration were also found to be higher in dyskinetic rats than non-dyskinetic rats, though plasma levels were not different (20), confirming this association. The availability of extracellular dopamine after exogenous levodopa administration depends significantly on the serotoninergic neurons, which convert it to dopamine and provide vesicular storage (21, 22). These neurons, however, lack the DAT system and pre-synaptic dopamine D2 autoreceptors, which leads to unregulated release and reduced clearance of dopamine (23). Denervation-dependent D1 receptor super-sensitivity causing pronounced activation of the D1-bearing striatal neurons (15) and changes in the dendritic and synaptic morphology (24) are two other important post-synaptic determinants of LID in animal models. Enhanced D1 receptor pathway transmission in dyskinetic animal models can lead to hyperphosphorylation of key enzymes necessary for neural signaling in the direct pathway (25). For example, GluR1, a subunit of AMPA receptors, exhibits high phosphorylation levels of Ser_831_ and Ser_845_ in the membranes of medium spiny neurons (MSNs) after levodopa treatment in dyskinetic rats (26). Thus, advanced PD is a state characterized by the inability to maintain stable, physiological, synaptic and extra-synaptic levels of dopamine, which in turn favors aberrant pre- and post-synaptic plastic responses in the striatum.

Here, we review the theories of LID related to striatal and cortical maladaptive plasticity in PD in the light of (1) the recent studies on motor cortex plasticity in different stages of evolution of PD (27–31), (2) the current knowledge of the physiology and anatomy of basal ganglia circuitry, including the reciprocal subcortical connections between the basal ganglia and the cerebellum (32, 33), and (3) the recent report of the role of cerebellar sensory processing in the bidirectional modulation of primary motor cortex (M1) plasticity (34) and the disturbance of this function in patients with LID (30). We propose a model based on the plasticity changes in PD that considers Parkinsonism, biphasic, and peak-dose dyskinesias as clinical manifestations of the abnormal interaction between M1 and the interlinked subcortical structures including the basal ganglia and cerebellum, during fluctuations in synaptic dopamine levels.

## Basal Ganglia Circuitry

### Cortico–basal ganglio–thalamo-cortical loop

**Figure 1:**
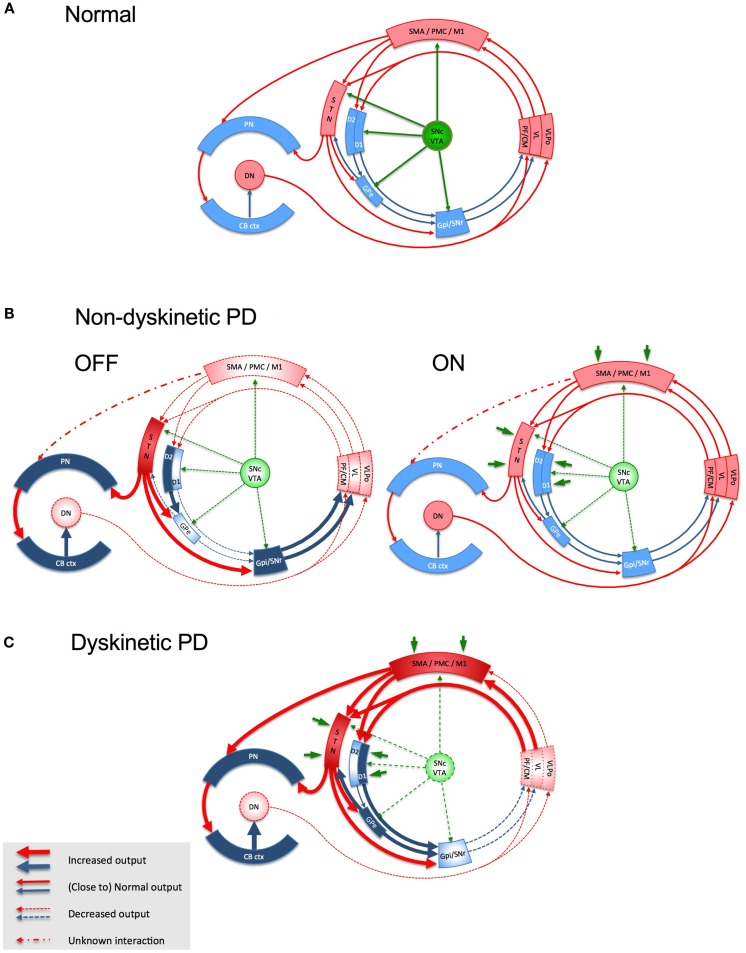
**Schematic representation of the basal ganglia- thalamo-cortical loop, the cerebello-thalamo-cortical loop and the interaction between the two in health (A), in non-dyskinetic Parkinson’s disease, after levodopa withdrawal (OFF) and after regular dose of levodopa (ON) (B), and in advanced Parkinson’s disease with levodopa-induced dyskinesia (C)**. Red arrows represent glutamatergic projections; blue arrows represent GABA-ergic projections; green arrows represent dopaminergic projections; dark green arrows in panel B and C represent the exogenous dopamine from levodopa. The shades of the blocks represent the activity of the respective network nodes. The STN is overactive because of cortical glutamatergic over activity during dyskinesias and from loss of GPe inhibition in OFF. The STN over activity locks cerebellar cortex in a persistent hyperactive state and interferes with its sensory processing function. The behavior of the cortico-ponto-cerebellar projections in non-dyskinetic PD in ON is not reported so far and is predicted by this model to be close to normal (CB ctx, cerebellar cortex; CM, centromedian thalamic nucleus; D1/D2, dopamine receptor types of the striatal medium spiny neurons (MSNs); DN, dentate nucleus; GPe, globus pallidus externus; GPi, globus pallidus internus; M1, primary motor cortex; PF, parafascicular nucleus; PMC, premotor cortex; PN, pontine nuclei; SMA, supplementary motor area; SNc, substantia nigra, pars compacta; SNr, substantia nigra, pars reticulata; STN, subthalamic nucleus; VL, ventro-lateral thalamic nucleus; VLPo, ventro-latero-posterior thalamic nucleus, pars oralis; VTA, ventral tegmental area).

Based on current understanding, the cortico–basal ganglia–cortical circuit functions as a complex, integrated network with multiple feed-back and feed-forward loops (35). The motor circuitry that projects from motor cortical areas (primary motor cortex, supplementary cortex, premotor cortex, and parts of the somatosensory dorsal parietal cortex) has a somatotopic, glutamatergic relay with the GABA-ergic MSNs in the dorsolateral portion of the post-commissural putamen and a small rim of the head of the caudate (36). The MSNs are connected to the output nuclei either indirectly, after relay in globus pallidus pars externa (GPe) and subthalamic nucleus (STN), or directly. The globus pallidus pars interna (GPi) and the substantia nigra pars reticulata (SNr) are the output nuclei of the basal ganglia and project to the premotor neurons in the ventral tier of thalamic nuclei (i.e., ventro-anterior, VA and ventro-lateral, VL), the centromedian (CM)/parafascicular (PF) complex, the pedunculo-pontine nucleus, the superior colliculus, and the brain stem (37–39). The VA/VL thalamic nuclei project to the supplementary motor area (SMA) and, to a lesser extent, to M1 and premotor cortex (38). In addition to the afferent input from putaminal MSNs and GPe neurons to STN, a hyper-direct glutamatergic pathway relays input from M1, SMA proper and pre-SMA, and dorsal and ventral premotor cortices to the dorsal aspect of STN (40–42). These hyper-direct glutamatergic cortico–STN connections, along with the STN–GPe and GPe–GPi connections, constitute the cortico–STN–pallidal pathways that bypass the striatum. The STN also receives glutamatergic projections from the thalamic PF and CM nuclei (43, 44). There are direct projections from STN to the cortex (45, 46) and to the thalamus (47). There are also dopaminergic projections from the SNc to STN (48, 49). Thus, dopamine can be seen to influence all glutamatergic synapses within the basal ganglia–thalamo-cortical circuit (Figure 1).

### Reciprocal basal ganglio–cerebellar circuitry

A major recent advance has been the identification of topographically organized, reciprocal links between basal ganglia and the cerebellum in non-human primates. Retrograde viral transporter studies in primates showed direct projection from the dentate nucleus, a major output of cerebellum, to the CM/PF complex intralaminar nuclei in the thalamus and subsequently to the dorsolateral putamen (32, 50). This bisynaptic path extends with a third synapse from putamen to GPe (32). It was recently shown in primates that the STN projects not only to the GPi, but also has topographically organized projections to lobule VII B and Crus II in the cerebellum via the pontine nuclei (51). The identification of bilateral subcortical communications between basal ganglia and cerebellum, besides their convergence to partially overlapping cortical areas (52–54), makes it imperative to examine the interaction between the abnormal basal ganglia activity and the cerebellar circuits in PD that could contribute to the development of Parkinsonism or LID.

## Dopaminergic Signaling in the Motor Circuits in Health

### Dopamine and basal ganglia

Dopaminergic fibers from the SNc innervate the striatum and all structures in the basal ganglia, including the STN and GPi, as well as the prefrontal, motor, and sensory cortices (55). Striatal synaptic and extra-synaptic dopamine levels are maintained at a constant level (56), independent of SNc neuronal firing, due to the efficient dopamine reuptake by the DAT system in the striatum (57) and the auto-inhibition mediated by presynaptic dopamine D2 receptor stimulation (58). The cortex and thalamus send massive glutamatergic input to the GABA-ergic striatal MSNs (59, 60) and dopamine has a pre-synaptic modulatory effect on this excitation (61). Post-synaptically, dopamine also stabilizes the firing rate and excitability of striatal neurons, inhibiting D2-bearing neurons and facilitating D1-bearing striato-pallidal neurons (62). Dopamine also regulates plasticity of striatal neurons by modulating glutamate-mediated long-term potentiation (LTP), long-term depression (LTD), and depotentiation (which is a homeostatic mechanism of reversal of a potentiated synapse to its pre-potentiated state) at the cortico-striatal synapses (63). Striatal LTP and LTD are important for motor learning, while depotentiation is thought to be necessary for removing unnecessary motor information (64). The activation of D1 receptors is necessary for induction of both LTP and depotentiation, while the co-activation of D1 and D2 receptors is required for induction of LTD. Thus, the midbrain dopaminergic neurons [SNc–ventral tegmental area (VTA)], through their projections to multiple basal ganglia nodes that receive glutamatergic inputs, can influence the local neural transmission as well as the induction of plasticity at these nodes (Figure 1). Dopamine can also indirectly influence M1 plasticity by regulating the basal ganglia inputs reaching M1.

### Dopamine and primary motor cortex

Neuroanatomical studies have confirmed the presence of direct dopaminergic innervations from the midbrain to M1 in rats (65, 66), monkeys (67), and human beings (68), as well as the presence of dopamine D1 and D2 receptors in M1 (69–72). A recent study, using the more specific DAT immunostaining method in mice, confirmed direct dopaminergic innervations of deep layers of M1 (73). D2 receptor agonists that increased the firing rate of pyramidal neurons proved the effect of dopamine on M1 neurons. This effect could be either due to a direct increase of pyramidal neuronal excitability or due to a reduction of inhibitory interneuronal activity (74). An important role of dopamine in M1 is to facilitate motor learning (66, 75) and motor memory encoding (76). In rats, the impaired LTP and motor skills learning following dopaminergic deafferentation of M1 could be corrected by local administration of levodopa within M1 using osmotic mini-pumps (66). However, denervation of SNc resulted in a total loss of motor skills learning, far more severe than direct M1 denervation (77). Vitrac et al. (73) showed that D1 receptors can enhance the associability of the pre- and post-synaptic activity: by increasing the sensitivity and the time-window for LTP induction, they serve as “coincidence modulators” that can determine whether a synapse will undergo LTP in response to a set of activity patterns. Dopamine is also considered necessary for the stability of motor representations within M1, as local injection of D2 receptor blockers results in the collapse of motor representations and of motor cortex excitability (78). This may be because both D1 and D2 receptor-mediated mechanisms within M1 are necessary for the intracortical horizontal connections to form LTP (75).

In human beings, studies in which plastic changes in M1 were artificially induced with non-invasive stimulation techniques (e.g., transcranial direct current stimulation, tDCS, and transcranial magnetic stimulation, TMS) revealed that dopamine has a non-linear dose-dependent effect on M1 plasticity (79–81), which is mediated through both D1 and D2 receptor subtypes (82). In healthy subjects, both low and excessive exogenous dopamine impair both facilitatory and inhibitory cortical plasticity (80, 83), while medium doses of oral levodopa have more stable effects and can even enhance performance in motor learning tasks (76).

### Dopamine and cerebellum

Animal studies revealed that there is a small but well-defined dopaminergic system in the cerebellum, which expresses all types of dopamine receptors and whose properties are similar to the striatal dopaminergic system (84). It receives inputs from the SNc and VTA that terminate in the granule and Purkinje cell layers (85–88). This system is important for the optimal development and functioning of both the cerebellum and the basal ganglia. Loss of nigral neurons in neonatal 6-OHDA-treated rats affects post-natal cerebellar development (89) and the expression of GABA_A_ receptor subtype (90). This highlights the dependency of cerebellar development on dopaminergic input, which could be direct or indirect through the basal ganglia. In addition, both the degeneration of Purkinje cells in a knock-out rat model (84) and kainic acid-induced degeneration of cerebellar cortex with preservation of deep cerebellar nuclei (91) led to up-regulation of D1 receptors and DAT in the striatum. This suggests that the cerebellar cortex, through the deep nuclei and the thalamic relay, down-regulates the striatal D1 receptors.

Besides the modulation of the striatal dopaminergic system, the cerebellum also regulates the cortical dopaminergic system, since electrical stimulation of dentate nucleus can induce dopamine release within the prefrontal cortex in mice (92). This might occur either through dentato-tegmental projections or through dentato-thalamo-cortical projections (93).

In healthy humans, fMRI studies have shown strong connectivity between SNc and cerebellum (94, 95). This connectivity between SNc and cerebellum is lost in PD but is restored by levodopa (96). Thus dopamine has both a direct and indirect influence on the cerebellum.

## Dopaminergic Signaling in Parkinsonism and Levodopa-Induced Dyskinesia

### Striatal signaling

According to the current models of basal ganglia in PD, dopamine depletion causes under-activation of GPe and disinhibition of STN in the indirect pathway (Figure 1B). STN over-activity in PD could not only be due to abnormalities of the indirect pathway, but also to the direct excitatory drive from the motor cortex and thalamus (97, 98). This could lead to excessive activation of GPi, a dysfunction favored also by the reduced input from D1 pathway. The net outcome would be enhanced GABA-ergic inhibition of thalamic projections to motor areas. It is now considered that not only firing rates but also firing patterns are important in maintaining normal signaling within the motor circuit. Intra-operative recordings in PD patients have demonstrated oscillations of local field potentials in the beta band in the STN that are synchronized with beta band oscillations in the cerebral cortex and GPi, indicating that excessive synchronization is a feature of the whole basal ganglia–cortical network. Dopaminergic drugs (99, 100) and STN DBS (101) can suppress the abnormal synchrony between basal ganglia and cortex. Moreover, suppression of beta band synchrony by these interventions positively correlates with improvement in bradykinesia and rigidity.

Levodopa treatment, however, does not restore basal ganglia activity to normal and LIDs are associated with reduced activity in the STN and GPi neurons. For example, peak oscillations at 4–10 Hz associated with dyskinesia were recorded only from the contralateral STN in patients with asymmetrical LID (102). Such abnormal oscillations in Parkinsonian and dyskinetic states could potentially propagate from the STN to the cerebellum in PD.

### Striatal plasticity in Parkinsonism and LID

In animal studies, striatal LTP and LTD are both impaired if dopaminergic afferents to striatum are lesioned but can be restored by levodopa (103). In human beings, the direct evidence of dopaminergic influence on an extrastriatal site was demonstrated in SNr neuronal plasticity during DBS surgery; local field potential amplitude of SNr neurons showed no enhancement after high frequency stimulation of STN when tested without levodopa, but enhancement was evident following the administration of levodopa (104).

In the rat models of PD and LID, striatal LTP was impaired in both non-dyskinetic and dyskinetic rats and could be restored by levodopa in both groups (64). In contrast, the capacity to depotentiate LTP was preserved only in non-dyskinetic rats. In the rat model, both the presence of dyskinesias and the loss of depotentiation were linked to higher doses of levodopa (105). This shows that loss of depotentiation at the cortico-striatal synapses from levodopa exposure is a marker of LID in animal models.

### Cortical signaling

A significant loss of dopamine and noradrenaline occurs in the motor cortex of PD patients (36, 55, 68). This postmortem finding was confirmed *in vivo* by PET of the motor cortex (106), motor cortical pathology was therefore proposed to underlie some of the symptoms of PD (106, 107). In line with this hypothesis, anti-PD drugs can modulate the activity of motor cortex (108) and non-invasive stimulation of the motor cortex can help reduce symptoms of PD and LID by reestablishing the homeostasis of some circuits (109). However, the kinetics of cortical dopamine within M1 is yet to be explored in human beings and animal models of LID.

### Motor cortex plasticity

**Figure 2:**
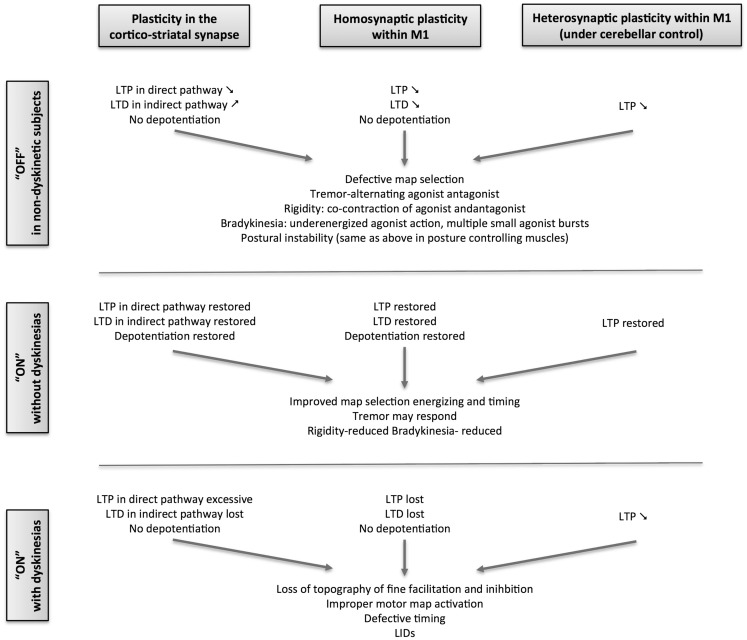
**Schematic overview of the evolution of plasticity in Parkinson’s disease from the stable, non-dyskinetic state to the dyskinetic state**.

In human PD, changes in motor cortex plasticity have been studied using transcranial magnetic stimulation (TMS), delivered alone as theta-burst (TBS) or as a paired associative stimulation (PAS), in which magnetic pulses delivered over M1 are classically associated with sensory pulses delivered on a peripheral nerve (Figure 2). The plastic effects of TBS depend on the stimulation pattern, which will act upon the intracortical synapses within M1. The sensorimotor plastic effects of PAS depend on the coincidence of pre- and post-synaptic neuronal activation within a specific time window (110–112). Using TBS in *de novo* PD, the LTP- and LTD-like plasticity of the intracortical circuits within M1was shown to be symmetrically and severely impaired even though the motor symptoms were unilateral (29). There was also no correlation of the plasticity loss with motor signs of PD, indicating that that M1 changes were more likely the direct consequence of mesocortical denervation than the indirect consequence of striatal denervation, the latter being more correlated with Parkinsonian signs. It also indicates that changes in M1 plasticity occur at a lower level of dopaminergic denervation than the higher threshold of 60–80% striatal dopaminergic denervation necessary for the motor signs to develop. Additionally, the local M1 plasticity in *de novo* patients did not show any short-duration response to a single dose of 100 mg levodopa, while clinical deficits showed significant improvement. This indicated that normalization of plastic mechanisms within M1 needs sustained dopamine replenishment. It was subsequently demonstrated that the propensity of intracortical circuits in the motor cortex to express normal plastic responses was closely linked to the stability of clinical response to levodopa (28):

(1) Patients with stable clinical response to levodopa with no fluctuations in their clinical response to levodopa and without LID could express LTP and LTD even when tested without levodopa. This suggests a beneficial and persistent treatment effect of levodopa therapy on M1 plasticity when compared to the severe loss of plasticity in the untreated state.
(2) Patients with wearing-off motor fluctuation showed a persistent treatment effect of chronic levodopa treatment on LTP, but not on LTD. In these patients, exposure to an acute boost of dopamine, by administering their regular dose of levodopa, revealed a detrimental effect of dopamine on both LTP and LTD.
(3) Patients with both motor fluctuations and dyskinesias have no persistent treatment effect on either LTP or LTD. Acute boost of levodopa did not restore LTP or LTD. Interestingly, acute dopamine boost led to a paradoxical LTP in the motor cortex of these patients in response to an intervention that normally induces LTD in healthy subjects. The paradoxical facilitation was more in those with more severe disease and with higher clinical response to levodopa, suggesting that both are consequences of the acute dopamine replacement. Moreover, PD patients with LID are unable to depotentiate an already established LTP in M1after an acute dosing with even a small dose of levodopa (113). This finding is similar to changes in the striatum of dyskinetic animals (64), but in contrast to non-dyskinetic patients in whom depotentiation was present after their regular dose of levodopa. PD patients with LID could express LTP in M1, but only when given half the regular dose of levodopa, reconfirming the negative effect of the regular dose on LTP. All these findings point to a severe dysregulation of intrinsic plastic mechanisms within M1 of patients with LID.

Additionally, studies using PAS have also revealed severe loss of sensorimotor, associative plasticity of M1 in the more affected hemisphere of *de novo* PD patients (31). Unlike the plastic response of the local intracortical circuits, the impairment in associative plasticity in the more affected hemisphere correlated with the severity of motor signs of PD, indicative of a dependence on striatal denervation underlying both. The short-duration response of associative plasticity to levodopa in *de novo* patients has not been studied so far. However, associative plasticity of M1can be restored by dopaminergic drugs only in chronically treated patients that are non-dyskinetic and not in dyskinetic ones (27).

Besides the plastic responses, intracortical inhibitory circuits are also altered in M1 of treated PD patients. GABA_A_-mediated, short-interval intracortical inhibition is reduced in PD patients, both dyskinetic and non-dyskinetic, when compared to controls, and levodopa could not correct this (114). In the same study, long interval intracortical inhibition was weaker than in controls in both non-dyskinetic and dyskinetic patients tested without levodopa, but levodopa could weakly correct this in dyskinetic patients. The lack of positive effect of levodopa on intracortical inhibition in M1 and the negative effect of levodopa on short latency afferent inhibition (115) has also been suspected to play a role in LID.

## Cerebellar Link to Motor Circuits in Health, Parkinsonism and LID

### Cerebellar plasticity in health

Within the motor control loops, the cerebellum controls and co-ordinates complex movements and is important for adapting movements to changes in feed-back. It receives sensory and motor information from descending cortical pathways and from ascending peripheral pathways. It has also connections to the parietal, premotor, and frontal cortices. The two major excitatory afferents to cerebellum are the climbing fibers and mossy fiber – parallel fiber systems, information from both of which eventually converges on the Purkinje cells that represent the only efferent output from the cerebellar cortex. The exteroceptive and proprioceptive inputs from the spinal cord and the input from pontine nuclei convey information from brainstem nuclei via mossy fibers to the granule cells. The axons of granule cells form the parallel fibers network. Climbing fibers originate in the inferior olive and relay directly to the Purkinje cells. Plastic changes in the strength of synapses relaying from the climbing and parallel fibers to the Purkinje cells are important in motor learning. Plasticity of climbing fibers input bi-directionally adjusts the plasticity of parallel fibers–Purkinje cells synapses. This suggests a role of climbing fibers as an error detector, which signals the need for adjusting the gain of sensory inputs and/or motor output within the cerebellum. Any disturbance in cerebellar plasticity could interfere with this function and result in maladjusted information delivered to M1, leading to abnormal, movement sequences.

### Cerebellar plasticity dysfunction in Parkinson’s disease and LID

There are several lines of evidence suggesting that cerebello–thalamo-cortical communication is abnormal in PD. In animal models of PD, the thalamic neurons that receive cerebellar output are underactive, just as those receiving basal ganglia afferents (116), indicative of a reduced dentato-thalamo-cortical excitatory output. In the MPTP mouse model, nigral degeneration is accompanied by loss of Purkinje cells in the cerebellum (117). In the untreated chronic MPTP monkey model of PD, nigral degeneration correlates with persistent hyperactivity in the cerebellum (118). Such a hyperactive state, secondary to striatal dopaminergic denervation, could prevent the efficient processing of the inputs from both parallel and climbing fibers, thus interfering with the plasticity mechanisms within the cerebellar cortex. One potential source of cerebellar hyperactivity could be the pathological hyperactivity in the glutamatergic projections from STN to cerebellum (Figures 1B,C). There is now increasing clinical, electrophysiological, and functional imaging evidence to invoke a cerebellar dysfunction in PD – for review see Ref. (119).

In the monkey model of PD, the ventro-lateral posterior nucleus of the thalamus that receives cerebellar output shows oscillations at tremor frequency, while in human PD, cerebellum has been linked to the postural tremor (120, 121). Also in PD patients, tremor-related abnormal oscillatory activity was recorded in the STN and GPi but not in the thalamic nuclei receiving basal ganglia input (122, 123). Considering the newly described anatomical connections between the basal ganglia and the cerebellum, it is conceivable that abnormal oscillations from a cerebellar circuit could propagate either to M1 and subsequently to STN via hyper-direct pathway, or from cerebellum to striatal MSNs projecting to GPe–STN via the indirect pathway. Defects in striatal dopamine release (124) and in cerebellar sensory processing function (125) were recently found in patients with primary focal dystonia. Abnormal signaling between basal ganglia and cerebellum in human PD could therefore potentially cause dystonic symptoms in untreated PD and during low plasma levels of dopamine as in biphasic dyskinesias.

Despite the classical view that LID might be generated exclusively by the disinhibition of cortical motor areas secondary to abnormal output from the basal ganglia in the striato–thalamo-cortical circuit (17), there is indirect evidence that the cerebellum may play a role in it.

In patients with PD, the binding potential of sigma receptors in the cerebellum (as explored with PET) is highly increased with respect to healthy controls and correlates only with LID scores and not with severity scores of Parkinsonian signs (126). Sigma receptor stimulation influences Purkinje cell firing (127) and also plays a role in the modulation of the glutamatergic/NMDA neurotransmission in the dopaminergic systems (128). Successful stereotaxic pallidal surgery (either pallidotomy or deep-brain stimulation) can lower this exaggerated sigma receptor binding in the cerebellum in LID (126).

Further evidence of cerebellar involvement in LID comes from TMS studies. In patients with PD, with mild to moderate LID, repeated sessions of bilateral cerebellar inhibitory stimulation after regular doses of levodopa induce a sustained reduction of dyskinesia lasting at least 2 weeks (30, 129). Repeated cerebellar stimulation can also reduce the cerebellar cortical activity and enhance dentate nuclear activity in imaging studies in PD patients with dyskinesias (130). In such patients, a single session of inhibitory stimulation of the cerebellar cortex combined with levodopa can restore the sensorimotor plasticity tested by PAS, but not the local intracortical plasticity as tested with TBS (30). This effect of cerebellar stimulation would have improved M1 plasticity induced by both PAS and TBS, if cerebellar stimulation was modifying directly M1 excitability. The lack of effects of cerebellar stimulation on intracortical plasticity of M1 and on intrinsic cortical excitability parameters (as reflected by the motor thresholds or intracortical facilitation and inhibition) supports a primarily subcortical mechanism. Recent studies showed that cerebellar stimulation can modulate the M1 associative heterosynaptic plasticity in healthy subjects (131) through the modulation of peripheral sensory afferents (34), eventually scaling the amplitude and topographic specificity of the associative plastic response (34); excitation of the posterior cerebellar cortex led to loss of associative plastic response, while inhibition of the cerebellar cortex led to prolonged facilitatory response to PAS with loss of topographic specificity. These were observed for PAS but not for TBS, suggesting that the target of cerebellar modulation is mainly the afferent input to M1 rather than M1 in itself. Cerebellar cortical excitation (i.e., heightened output of the Purkinje cells) leads to the enhancement of the normal inhibition of the dentate nucleus, which would reduce the normal excitatory control of dentate nucleus on the afferent inflow to M1, probably at the thalamic or olivary nuclear level, thus blocking the sensorimotor-plasticity within M1. In contrast, cerebellar cortical inhibition (i.e., depressed output of the Purkinje cells) could lead to disinhibition of dentate nucleus, which would facilitate afferent input to M1 (34). This model is in keeping with the adaptive filtering role of the cerebellum on sensory afferents (132). Additionally, repeated sessions of inhibitory stimulation of the posterior cerebellar cortex can restore the levodopa-unresponsive, associative M1 plasticity in dyskinetic PD patients, concurrent with the reduction in LID severity (30). Several findings of this particular study suggested that the reduction in LID could be related to the improvement of responsiveness of M1 to PAS after cerebellar inhibition: (1) larger facilitation of M1 plasticity after a single session of cerebellar inhibitory stimulation in ON predicted greater anti-dyskinetic effect of repeated cerebellar stimulation in the same subjects; (2) the time course of LID improvement was similar to the time-course of the associative plasticity restoration after 10 consecutive sessions of cerebellar stimulation; and (3) patients with more severe LID before the TMS treatment showed larger responsiveness of M1 to cerebellar inhibition suggesting more involvement of the cerebellum in the pathophysiology of dyskinesias as the severity of LID increases. However, cerebellar stimulation did not worsen or enhance the Parkinsonian signs in OFF or beyond the effect of levodopa alone. Patient diaries revealed that the time spent in ON without troublesome dyskinesias, but not the durations of the OFF periods, was improved by cerebellar stimulation. These suggest that combined levodopa replacement therapy and cerebellar inhibition might be required to restore the balance between the two circuits and to concurrently improve Parkinsonian signs and reduce dyskinesias. Exogenously derived dopamine might act by increasing the excitability of M1 neurons (73) and normalizing basal ganglia signaling, whereas inhibition of the cerebellar cortex enhances the gain of the sensory afferent input and allow better sensorimotor integration (34). It remains speculative whether cerebellar hyperexcitation exists in *de novo* human PD as in *de novo* animal model of PD and if this cerebellar defect contributes to the genesis of specific manifestations like tremor or dyskinesias. If it were the case, then this metaplastic state of the cerebellar cortex could be reversed or delayed by early artificial inhibitory stimulation of the cerebellar cortex.

Earlier neuroimaging studies have found increased activity of motor and premotor areas in dyskinetic PD patients when compared to non-dyskinetic patients (133, 134). This led to the classical view that hyperactivity in cortical motor areas might be responsible for LID (17, 135). However, inhibitory stimulation of SMA (135, 136) or of M1 (137) failed to provide sustained improvement of dyskinesias. The results obtained recently with cerebellar stimulation point to an alternative explanation, which reconciles with the observations in these older studies: as SMA, pre-motor cortex, and M1 are all targets of cerebellar output (54). An abnormal input from the cerebellum to SMA or M1 in dyskinetic patients might indeed trigger abnormal fMRI activations, but only as downstream, secondary, phenomena, thus making them unsuitable targets for a direct intervention for the treatment of LID.

## Proposed Model of Parkinsonism and Levodopa-Induced Dyskinesia Based on Aberrant Plasticity

This model is based on the view that a physiological level of dopaminergic stimulation is critical for maintaining normal plasticity at the glutamatergic terminals in the interconnected large motor network involving the basal ganglia, motor cortices, thalamus, and sensorimotor areas of the cerebellum. This model considers M1 only as the final target of this network and proposes LID as the product of a cascade of changes triggered by the altered dopaminergic signaling in this network. M1 neurons deprived of the ability to depotentiate activated synapses due to abnormally high synaptic dopamine levels during peak-dose, are rendered indiscriminately receptive to non-salient or even aberrant afferent inputs from the environment conveyed by other neural structures (spino-thalamic pathways via ventro-lateral thalamic nuclei, other cortical areas via direct cortico-cortical connections, etc.). This defect may be additionally amplified by the aberrant signals reaching M1 from the thalamic relays erratically modulated by the basal ganglia and cerebellum (Figure 1C). The result would be the inappropriate selection of motor programs and the generation of movements that are both unwanted and abnormal. The absence of effective inhibitory control within M1 and the paradoxical facilitation during attempted inhibition would make these movements resistant to voluntary suppression and even exacerbate them. Biphasic dyskinesia co-existing with Parkinsonism may reflect transitory oscillations in the synaptic dopamine levels during the rising and falling phases of release of exogenously derived dopamine. Such oscillations might allow concomitant manifestations of severe Parkinsonism and biphasic dyskinesias. This model needs further experimental validation. It also raises many questions that could be tested in future studies:

(1) Does DBS of the STN influence both STN–thalamo-cortical and STN–cerebellar transmission? Though the exact mechanisms by which DBS of the STN improves PD are not fully established, the fact that effective DBS of the STN improves cerebellar hyperactivity (138, 139) suggests that it may influence both the STN–GPi and the STN–cerebellar projections. Extracellular recordings in MPTP-intoxicated primates have shown that during DBS of the STN, significant change occurs in the pattern of neuronal activity in areas of the motor thalamus receiving both pallidal and cerebellar projections (140).
(2) The dose of administered levodopa has to be reduced after immediately after DBS of the STN to reduce dyskinesia. Does this indicate that DBS of the STN by itself cannot normalize the aberrant plasticity within M1 caused by dopamine surges, which would be necessary to alleviate dyskinesia? Preliminary data showing that sensorimotor cortex plasticity improves only after long-term synergistic combination of DBS with reduced doses of levodopa, but not after DBS alone (141). That long-term stimulation is required for restoring sensory afferent inhibition of M1(142) indicates that cerebellar control of sensory processing may also normalize only after chronic stimulation of STN.
(3) Does selective DBS of the ventral GPi but not of the dorsal GPi or STN simultaneously inhibit the propagation of pro-dyskinetic signals through both cortico-striatal synapses and cerebello-striatal synapses (relayed through CM/PF thalamic nuclei) on D1-bearing MSNs? It is already known that GPi has functional somatotopy (143) and that ventral GPi stimulation has anti-dyskinetic effects while dorsal GPi stimulation improves akinesia and induces dyskinesias (144). In primates, tightly connected functional circuits have been described between basal ganglia and the CM/PF, with a sensorimotor circuit linking the post-commissural putamen, the centro-lateral part of the caudal GPi, and the medial two-thirds of the CM nucleus (145). The fact that CM/PF is a target for both pallidal and cerebellar inputs (39) might then explain why DBS of CM/PF is useful for controlling tremor that is resistant to DBS of the STN and also LID that are only partially responsive to DBS of GPi. However, DBS alone of the CM/PF does not change global UPDRS as strongly as DBS of the STN or GPi (146).

## Conflict of Interest Statement

The authors declare that the research was conducted in the absence of any commercial or financial relationships that could be construed as a potential conflict of interest.
